# Evaluation of Functional Outcomes and Quality of Life in Elderly Patients (>75 y.o.) Undergoing Minimally Invasive Radical Cystectomy with Single Stoma Ureterocutaneostomy vs. Bricker Intracorporeal Ileal Conduit Urinary Diversion

**DOI:** 10.3390/jcm11010136

**Published:** 2021-12-27

**Authors:** Andrea Fuschi, Yazan Al Salhi, Manfredi Bruno Sequi, Gennaro Velotti, Alessia Martoccia, Paolo Pietro Suraci, Silvio Scalzo, Anastasios Asimakopoulos, Giorgio Bozzini, Alessandro Zucchi, Cosimo De Nunzio, Antonio Carbone, Antonio Luigi Pastore

**Affiliations:** 1 Urology Unit, Department of Medico-Surgical Sciences and Biotechnologies, Faculty of Pharmacy and Medicine, Sapienza University of Rome, 04100 Latina, Italy; andreafuschi@gmail.com (A.F.); yazan5585@gmail.com (Y.A.S.); mb.sequi@gmail.com (M.B.S.); gennaro.vel88@gmail.com (G.V.); martoccia.alessia@gmail.com (A.M.); spaolopietro@gmail.com (P.P.S.); silvioscalzo@hotmail.it (S.S.); antonio.carbone@uniroma1.it (A.C.); 2 ICOT-Surgery, Orthopedics, Traumatology Institute, 04100 Latina, Italy; 3 Urology Unit, Tor Vergata University, 00100 Rome, Italy; tasospao2003@yahoo.com; 4 ASST Lariana, 22100 Como, Italy; gioboz@yahoo.it; 5 Department of Urology, University of Pisa, 56121 Pisa, Italy; zucchi.urologia@gmail.com; 6 Department of Urology, Faculty of Medicine and Psychiatry, Sant’Andrea Hospital, 00100 Rome, Italy; cosimodenunzio@virgilio.it

**Keywords:** bladder cancer, elderly patient, urinary diversion, heterotopic ileal conduit, Bricker, ureterocutaneostomy, single stoma, robot-assisted, laparoscopy, QoL Stoma instrument

## Abstract

Background: Diversion after radical cystectomy (RC) is crucial when considering elderly subjects. Data on the quality of life (QoL) impact with different diversions is scarce. This study aims to compare complications and QoL in patients aged > 75 y.o., who underwent minimally invasive (MI) RC with Bricker intracorporeal urinary derivation and single stoma ureterocutaneostomy. Methods: We conducted a retrospective analysis of elderly patients who underwent MIRC and intracorporeal diversion. The 78 subjects were divided into two groups: group A, ileal conduit, and group B, single stoma ureterocutaneostomy. We evaluated the bowel’s recovery time and complications rate. We investigated QoL 3 and 6 months after surgery using the Stoma-QoL questionnaire. Results: Mean age was 77.2 in group A and 82.4 in group B. The mean ASA score and Charlson Comorbidity index were comparable between the two groups. Rates of complications were 57.6% and 37.4% in groups A and B, respectively. The mean postoperative Stoma-QoL score 3 months after surgery was 52.2 and 52.4 in groups A and B, respectively. At 6 months of follow-up the Stoma QoL mean score was 63.4, showing homogeneity between the groups. Conclusion: MIRC with single stoma ureterocutaneostomy represents an alternative to ileal conduit, with comparable QoL and ostomy management 6 months after surgery, reporting fewer complications.

## 1. Introduction

Radical cystectomy (RC) with lymphadenectomy represents the “gold standard” treatment for muscle-invasive and selected high-risk non-muscle-invasive bladder cancer [1,2,3]. However, this surgical procedure is frequently associated with perioperative morbidity, especially in elderly patients with relevant comorbidities. Complications observed after RC in these patients can be directly related to the performed urinary diversion [2]. During radical cystectomy, the reconstructive time, using intestinal loops for the reconfiguration of the urinary diversion, exposes patients to medium and/or severe complications in accordance to Clavien–Dindo classification [4]. On the other hand, ureterocutaneostomy (UCS) can be performed quickly, significantly reducing operative time and the potential risk of intestinal and metabolic complications compared to the ileal conduit. This is an essential factor to consider when evaluating geriatric patients with advanced disease and limited life expectancy [2]. Technically, in most cases, ureterocutaneostomy is performed bilaterally, creating two ostomies, which could negatively affect patient body image and quality of life [5]. Consequently, the choice of urinary diversion is a key factor to consider when planning radical cystectomy. However, little to no information is available in the literature regarding the complications and impact on patients’ quality of life (QoL) associated with the different urinary diversions, especially in elderly patients. This study aims to compare perioperative and postoperative complications and QoL in two groups of patients aged > 75 and in a high comorbidity state, who underwent minimally invasive (laparoscopic or robot assisted approach) radical cystectomy with intracorporeal ileal conduit (Bricker) or single stoma ureterocutaneostomy.

## 2. Materials and Methods

The study was conducted as a retrospective analysis of the medical records of patients with muscle-invasive bladder cancer (MIBC) and high-risk non-muscle-invasive bladder cancer (NIMBC), who underwent laparoscopic or robot-assisted RC with intracorporeal ileal conduit (as described by Bricker) or single stoma ureterocutaneostomy. Medical records from May 2016 to March 2020 were analyzed and divided into two study groups in relation to the urinary diversion performed: group A included patients who underwent Bricker ileal conduit reconstruction, while group B included patients with single stoma ureterocutaneostomy.

The studied population was adequately assessed for age, BMI, smoking habit, neurological pathologies, American Society Anesthesiologists classification (ASA score), Charlson Comorbidity Index (CCI), urinary continence, and erectile function by the International Index of Erectile Function questionnaire (IIEF-5) [2]. In addition, the complete evaluation included: preoperative blood estimated glomerular filtration rate (eGFR), hemoglobin values (Hb), and preoperative pathological reports at the time of transurethral resection of the bladder (TURB).

The data that was collected and analyzed were: operative time (min), Hb (gr/dL) after 24 h, and postoperative eGFR 1 month after the surgical procedure, as well as the rate of intra- and postoperative blood transfusion, hospitalization in postoperative intensive care, bowel recovery time, neoadjuvant chemotherapy, pathological report, lymph node involvement, rate of complications according to Clavien–Dindo classification, conversions to open surgery, and assessment of QoL using the Stoma-QoL instrument at 3 and 6 months after the surgical procedure [2,4,6].

Several questionnaires have been validated to evaluate QoL in patients with bladder cancer, including FACT (Functional Assessment of Cancer Therapy) [7], EORTC QLQ C-30 [8], EORTC QLQ-BLM (a muscle-invasive bladder cancer module) [9], and SF (Short Form)-36 [10,11] and recently the Bladder Cancer Index (BCI) questionnaire designed and validated specifically for patients with bladder neoplasia [12]. Among these, the “Questionnaire to assess the quality of life among ostomy people”, Stoma-QoL, represents an important instrument to evaluate the significant problems concerning intimate relationships, relationships with family and friends, and the main difficulties of managing the ostomy during daily routine. This questionnaire consists of 20 items, is easy to complete, provides a general overview of the patients’ quality of life, and turns out to be a valid test that provides reliable results [5].

### 2.1. Bricker Ileal Conduit Diversion

The same surgical steps were performed in both minimally invasive approaches (laparoscopic and robot assisted). After RC with LND, an ileal segment of about 10–15 cm in length was identified, 20 cm from the ileocecal valve. The left ureter was transposed under the colon sigma and above the great vessels on the right side. If the length of the left ureter was short, a sigmoid retraction was performed. After creating two mesenteric windows using a laparoscopic hook or robotic monopolar scissors, a 45 mm Endo GIA automatic stapler was used to dissect the bowel proximally and distally. The distal ends obtained were held together using a 3-0 self-cinching barbed suture to avoid malrotation during the anastomosis (Marionette stitch). Then, anastomosis of the two intestinal stumps was performed using an automatic suturing machine. Two openings were made on either side of the proximal end of the duct using scissors or a monopolar hook. Next, spatulation of the distal end of both ureters was performed. Uretero-ileal anastomosis was performed according to Bricker’s technique; an end-to-side anastomosis keeping the anastomoses separate was performed as well.

The posterior wall of the uretero-ileal anastomosis was completed using a 3-0 barbed self-cinching suture, and a 6 or 8 Fr. mono-J ureteral stent was placed bilaterally. The stents were fixed to the ureters using a 3-0 Vicryl rapid suture.

The ostomy was placed on the middle third of the line between the right anterior superior iliac spine and the umbilicus.

### 2.2. Single Stoma Ureterocutaneostomy

The ureters were gently mobilized to preserve the blood supply as far as possible. A single stoma ureterocutaneostomy was performed, incising the skin about 4 cm below the umbilicus, and the ends of the ureters were passed through the abdominal wall by soft grasper forceps. The left ureter was transposed to avoid tensions behind the mesosigmoid and was positioned in front of the great vessels and through the opening in the retroperitoneal region on the stomal side. Both ureters were fixed to the abdominal fascia using a Vicryl 3-0 suture. Then, the ureters were spatulated to the same length and were splinted with 6 or 8 Fr mono-J ureteral stents. The medial walls of the mucosa of both ureteral flaps were anastomosed, and the free edges of the ureters newly conjoined were fixed to the cutaneous layer by 4 circumferential stitches performed using a Vicryl rapid 3-0 suture.

### 2.3. Statistical Analysis

Statistical analysis was preformed using the Statistical Package for the Social Sciences (SPSS), version 25.0 (SPSS Inc., Chicago, IL, USA). All *p*-values < 0.05 were considered statistically significant. The t-test was used to compare means of independent groups, while a two-tailed t-test performed the comparison of continuous variables between dependent samples for paired samples with 95% confidence intervals. The local ethics committee approved the study, and it was performed following the ethical standards of the 1964 Declaration of Helsinki and its later amendments. All patients gave their informed consent before their data collection and inclusion in the study.

## 3. Results

**Figure 1:**
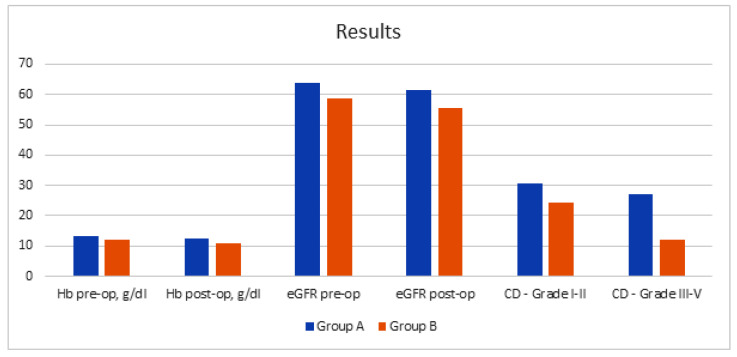
Intraoperative and postoperative results. Hb: Hemoglobin; eGFR: Estimated Glomerular Filtration Rate.

A retrospective analysis of 78 medical records of patients who underwent mini-invasive radical cystectomy with intracorporeal non-continent heterotopic neobladder (ileal conduit as described by Bricker) or single stoma ureterocutaneostomy, was conducted. According to the urinary diversion performed, patients were divided into two groups: group A, consisting of 37 patients (25 males and 12 females), who underwent a Bricker ileal conduit; group B consisted of 41 patients (28 males and 13 females), who underwent single-stomal ureterostomy (as double-barreled ureterocutaneostomy). The study was conducted on a total of 78 patients (53 males and 25 females, ratio: 2.12:1) divided into two statistically homogeneous groups concerning demographic data and preoperative characteristics, as reported in Table 1 and Figure 1.

The mean age was 77.2 years old (95% CI: 75.1–79.3; ES: 0.97) in group A and 82.4 (95% CI: 80.2–84.6; ES: 0.91) years old in group B (*p* value: 0.0005), both statistically homogeneous for BMI (mean group A: 24.1; ES: 0.64 vs. mean group B: 23.2; ES: 0.60; *p* value: 0.892). The mean ASA score was 2.4 (ES: 0.08) in group A and 2.8 (ES: 0.075) in group B (*p* value: 0.029), with a mean Charlson Comorbidity Index of 5.3 (ES: 0.11) and 6.4 (ES: 0.09), respectively (*p* value: 0.014) (Table 1 and Figure 1). The mean preoperative hemoglobin values (mean A: 13.4 g/dL; ES: 0.23; vs. mean B: 12.1 g/dL; ES: 0.28; *p* value: 0.084) and eGFR (mean A: 63.8; ES: 4.2 vs. mean B: 58.58; ES: 4.0; *p* value: 0.375) were lower in group B, but not statistically significant. The mean postoperative hemoglobin values at 24 h (mean A: 12.4 g/dL; ES: 0.27; vs. mean B: 10.9 g/dL; ES: 0.31; *p* value: 0.071) and eGFR at 1 month (mean A: 61.4; ES: 3.8 vs. mean B: 55.52; ES: 3.7; *p* value: 0.213) resulted lower in group B, so no statistical significance was even observed (Table 1 and Figure 1). There were 33% of patients in group A and 30% in group B that underwent neoadjuvant chemotherapy (*p* value: 0.311). The mean total operative time was statistically longer in group A compared to group B, 334 min (95% CI: 313–357; ES: 4.15) vs. 186 min (95% CI: 158–214 min; ES: 3.89), respectively (*p* value <0.0001). The intraoperative transfusion rate was 3.8% in group A and 4.2% in group B (*p* value: 0.064); blood transfusion was required postoperatively in 6.8% and 8.4% of patients in groups A and B, respectively (*p* value: 0.041; Table 1 and Figure 1).

The mean hospital stay in intensive care did not show significant differences between the two groups (1.4 days; ES: 0.07; vs. 1.6 days; ES: 0.05 days; *p* value: 0.151). However, the mean hospital stay was statistically higher in group A (mean: 9.2 days; ES: 0.67; vs. 6.5 days; ES: 0.55; *p* value: 0.002). Bowel function recovery was significantly faster in the UCS group than in group A (A: 3.6 days; ES: 0.12; vs. B: 1.5 days; ES: 0.04; *p* value: 0.0043). The evaluation of the TURBT pathological characteristics did not reveal statistically significant differences between the two groups, identifying in group A 19% of high-risk NMIBC cases and 81% of MIBC cases and in group B 11% of high-risk NMIBC cases and 89% of MIBC cases, respectively (*p* values > 0.05). Final histopathological examination showed 41.3% of pT3-pT4 in group A and 54.2% of pT3-pT4 in group B (*p* value: <0.0001). There were no statistically significant differences concerning lymph node involvement rate, even though a higher involvement in group B was reported (14.2% vs. 17.1%; *p*-value: 0.067; (Table 1 and Figure 1)).

**Figure 2:**
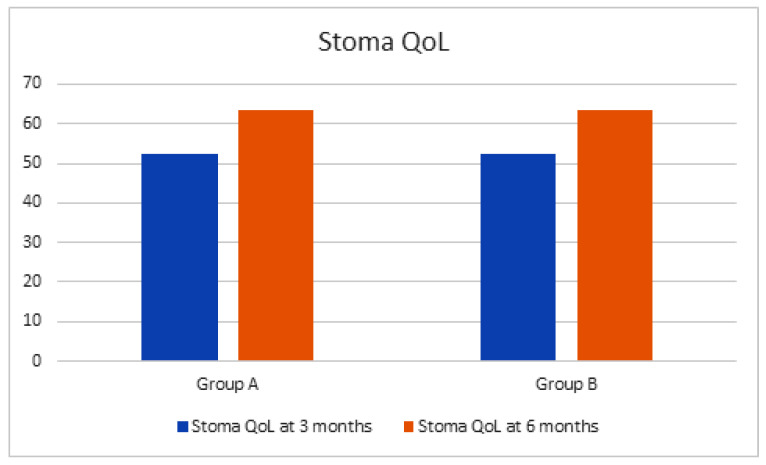
Stoma Quality of Life at 3 and 6 months of follow-up after surgery. QoL: Quality of Life.

Regarding perioperative and postoperative complications, per the Clavien–Dindo classification, 57.6% of patients in group A and 37.4% of patients in group B reported these results: grade I–II: 30.5% vs. 24.3%; grade III–V: 27.1% vs. 12.1% (*p* values: < 0.05). The most common complications in group A were represented by uroperitoneum (9.4%), ileus anastomosis dehiscence (6.1%), paralytic ileus (4.6%), and ureteral detachment (2.2%). The complications highlighted in group B were represented by uroperitoneum (6.3%) and paralytic ileus (1.8%). No deaths were recorded in the perioperative period nor at 90 days after the surgical procedure. Mean follow-up was 14.4 months (range 8–16 months). The postoperative assessment of QoL using the Stoma-QoL instrument showed in group A and B at 3 months of follow-up a mean score of 52.2 (95% CI: 49.4–55.0; ES: 1.61) and 52.4 (95% CI: 50.3–54.5; ES: 1.62), respectively. Furthermore, statistically homogeneous scores between groups even at 6 months of follow-up were observed, with a mean score of 63.4 (95% CI: 59.2–67.6; ES: 1.84) and 63.6 (95% CI: 61.3–65.9; ES: 1.82) in groups A and B, respectively (*p* values > 0.05; Figure 2).

## 4. Discussion

The assessment of the patient should be performed during the preoperative counseling to improve the decision-making process. Elderly patients with indications of radical cystectomy for bladder cancer represent a progressively increasing epidemiological relevance [13]. In this population, the choice of not performing radical surgery is directly related, in most cases, to disease progression and significant reduction of cancer-specific survival [13,14,15,16,17]. To date, several studies have reported their experience of treating elderly patients with bladder cancer with minimally invasive radical cystectomy with particular attention to the urinary diversion. However, few studies focused on the evaluation of the related QoL in these patients [13,18,19]. To date, only one study evaluated urinary diversion (Bricker and UCS) regarding perioperative and postoperative complications and post-surgical management [9]. Both techniques result in a single ostomy in the abdomen and, therefore, the need of external urine collection bags, unlike a classic bilateral ureterocutaneostomy, which results in two ostomies with a greater impact on the patient’s QoL. The UCS represents the simplest and less invasive form of urinary diversion without bowel manipulation. The main complication related to UCS is represented by stomal stenosis, with a higher incidence than ileal conduit and the need of UCS ureteral lifetime stenting [13]. The Bricker conduit does not require permanent ureteral stents, but complications related to the manipulation of the intestinal loops are frequent. In the literature, the incidence of complications after RC in the elderly varies from 28% to 64% [20], and most complications (41–72%) are medical [20]. Surgical complication rates range from 8% to 35% [20]. The wide variations in the frequency and spectrum of reported complications are mainly attributable to the heterogeneity of the postoperative evaluation period considered.

The present study shows better perioperative and postoperative results in the group that underwent UCS with single ostomy compared to patients undergoing ileal conduit, confirming the data already reported [21,22]. The other important result regarding quality of life shows that in both groups, no statistically significant difference was observed at 3 and 6 months of evaluation, and, therefore, in the management of single stoma.

In our study, a longer overall operating time was shown in the group of patients that underwent ileal conduit reconstruction compared to patients with UCS. However, no statistically significant differences were found between groups regarding blood loss, transfusion rate, eGFR, and intraoperative complications. Although not statistically significant, the transfusion rate was higher in group B than in group A; this result is attributable to the patients’ health status and to the preoperative comorbidities of patients undergoing UCS compared to ileal conduit group [13,21,22,23].

Relevant data are represented by the rate of perioperative and postoperative complications (according to Clavien–Dindo classification) that were higher in group A. This result might be attributable to bowel manipulation, ileal anastomoses, longer operative time, and greater exposure to pneoperitoneum in patients undergoing ileal conduit [13,21,22,23]. In addition to this, the increased use of anesthetic drugs is related to a slower bowel function recovery in patients undergoing ileal conduit than patients with UCS [13,21,22]. In accordance with the literature, despite the higher ASA score of patients in group B compared to group A, there was no statistically significant difference in relation to the mean stay in postoperative intensive care and the overall hospitalization length. In our study, the lower incidence of perioperative and postoperative complications reported was comparable to the literature; this could be partly explained by the exclusion of patients with history of previous pelvic surgery and radiotherapy [24]. Before 1990, perioperative mortality after RC was very high and ranged from 2.4% to 15%, but to date this incidence appears to be remarkably reduced as evidenced in the literature (0–3.9%) and confirmed in our study with no cases of conversion to open surgery and no mortality at 30 days after surgery [23]. Perioperative mortality in elderly patients undergoing RC with ileal conduit or UCS has been reported to vary between 0% and 10% [24]. The hospital stay in the present study agreed with the data found in the literature and, therefore, was statistically lower in the group of patients undergoing UCS compared to patients with Bricker heterotopic urinary diversion [13,25,26].

As shown in the literature, the QoL varies between the different urinary diversions, but no study has definitively demonstrated the superiority of one derivation over another in terms of QoL in elderly patients [27].

Our results showed an overlap of the questionnaire scores when evaluating patients’ compliance in the management of the single ureterocutaneostomy or intestinal ostomy during daily routines. Patients in both groups showed satisfaction, and the simplicity of managing a single ostomy from the analysis of the scores obtained, without complications, is in line with the data in the literature [28]

The study has several limitations. The follow-up period and the retrospective nature of the study may limit the observed results. However, the patients’ investigation through a Stoma-QoL questionnaire represents an important point of strength of this study. To date, this is the first study that compares the two most diffused heterotopic derivations in terms of quality of life and the daily routinary management of the ostomy.

## 5. Conclusions

In conclusion, in elderly patients this study shows that, for bladder cancer, the choice of minimally invasive radical cystectomy with single stoma ureterocutaneostomy could represent a valid alternative to heterotopic urinary diversion with Bricker ileal conduit, which is the more common alternative. This consideration stems, as evidenced from the obtained results, from the reduced rate of perioperative and postoperative complications, reduced operative time, faster recovery of bowel function, and lower hospital stay in patients who underwent UCS with single ostomy. Moreover, single stoma UCS was associated with a quality of life and management of the ostomy comparable to patients undergoing ileal conduit.

## Figures and Tables

**Table 1:** Preoperative and post-operative results.

	Group A	Group B	*p* Value
Patients, n. (m–f)	37 (25–12)	41 (28–13)	
Age, y.o. (ES)	77.2 (0.97)	82.4 (0.91)	0.0005
BMI, kg/m^2^ (ES)	24.1 (0.64)	23.2 (0.6)	0.892
ASA, (ES)	2.4 (0.08)	2.8 (0.075)	0.029
CCI, (ES)	5.3 (0.11)	6.4 (0.09)	0.014
Hb pre-op, g/dL (ES)	13.4 (0.23)	12.1 (0.28)	0.084
Hb post-op, g/dL (ES)	12.4 (0.27)	10.9 (0.31)	0.071
eGFR pre-op, (ES)	63.8 (4.2)	58.58 (4)	0.375
eGFR post-op, (ES)	61.4 (3.8)	55.52 (3.7)	0.213
Neoadjuvant CH, %	33	30	0.311
Total Operative Time, min (ES)	334 (4.15)	186 (3.89)	<0.0001
Intra-op blood transfusion, %	3.8	4.2	0.064
Post-op blood transfusion, %	6.8	8.4	0.041
Intensive Care, days (ES)	1.4 (0.07)	1.6 (0.05)	0.151
Hospital Stay, days (ES)	9.2 (0.67)	6.5 (0.55)	0.002
Bowel function recovery, days (ES)	3.6 (0.12)	1.5 (0.04)	0.0043
Lymphnode involvement, %	14.2	17.1	0.067
Clavien–Dindo Classification, %			
Grade I–II	30.5	24.3	0.003
Grade III–V	27.1	12.1	0.0041
Stoma QoL at 3 months, (ES)	52.2 (1.61)	52.4 (1.62	0.316
Stoma QoL at 6 months, (ES)	63.4 (1.84)	63.6 (1.82)	0.382

BMI: Body Mass Index; ASA: American Society of Anesthesiologists; CCI: Charlson Comorbidity Index; Hb: Hemoglobin; eGFR: Estimated Glomerular Filtration Rate; CH: Chemotherapy; QoL: Quality of Life.

## Data Availability

The data presented in this study are available on request from the corresponding author. The data are not publicly available due to privacy restrictions.

## References

[1] Kirkali Z., Chan T., Manoharan M., Algaba F., Busch C., Cheng L., Kiemeney L., Kriegmair M., Montironi R., Murphy W.M. (2005). Bladder cancer: Epidemiology, staging and grading, and diagnosis. Urology.

[2] Witjes J.A., Bruins H.M., Cathomas R., Compérat E., Cowan N.C., Gakis G., Hernández V., Lorchet A., Ribal M.J., Thalmann G.N. (2021). EAU Guidelines on Muscle-Invasive and Metastatic Bladder Cancer. Eur. Assoc. Urol..

[3] Babjuk M., Burger M., Compérat E.M., Gontero P., Mostafid A.H., Palou J., van Rhijn B.W.G., Roupret M., Shariat S.F., Sylvester R. Guidelines on Non-muscle-invasive bladder cancer (Ta, T1 and CIS). Proceedings of the 36th EAU Congress.

[4] Clavien P.A., Barkun J., de Oliviera M.L., Vauthey J.N., Dindo D., Schulick R.D., de Santibañes E., Pekolj J., Slankamenac K., Bassi C. (2009). The Clavien-Dindo classification of surgical complications: Five-year experience. Ann. Surg..

[5] Porter M.P., Penson D.F. (2005). Health related quality of life after radical cystectomy and urinary diversion for bladder cancer: A systematic review and critical analysis of the literature. J. Urol..

[6] Prieto L., Thorsen H., Juul K. (2005). Development and validation of a quality-of-life questionnaire for patients with colostomy or ileostomy. Health Qual. Life Outcomes.

[7] Cella D.F., Tulsky D.S., Gray G., Sarafian B., Linn E., Bonomi A., Silberman M., Yellen S.B., Winicour P., Brannon J. (1993). The Functional Assessment of Cancer Therapy scale: Development and validation of the general measure. J. Clin. Oncol..

[8] Aaronson N.K., Ahmedzai S., Bergman B., Bullinger M., Cull A., Duez N.J., Filiberti A., Flechtner H., Fleishman S.B., De Haes J.C.J.M. (1993). The European Organization for Research and Treatment of Cancer QLQ-C30: A quality-of-life instrument for use in international clinical trials in oncology. J. Natl. Cancer Inst..

[9] Sogni F., Brausi M., Frea B., Martinengo C., Faggiano F., Tizzani A., Gontero P. (2008). Morbidity and quality of life in elderly patients receiving ileal conduit or orthotopic neobladder after radical cystectomy for invasive bladder cancer. Urology.

[10] Ware J.E., Sherbourne C.D. (1992). The MOS 36-item short-form health survey (SF-36). I. Conceptual framework and item selection. Med. Care.

[11] Ware J.E., Keller S.D., Gandek B., Brazier J.E., Sullivan M. (1995). Evaluating translations of health status questionnaires. Methods from the IQOLA project. Int. J. Technol. Assess. Health Care.

[12] Gilbert S.M., Dunn R.L., Hollenbeck B.K., Montie J.E., Lee C.T., Wood D.P., Wei J.T. (2010). Development and validation of the Bladder Cancer Index: A comprehensive, disease specific measure of health-related quality of life in patients with localized bladder cancer. J. Urol..

[13] Deliveliotis C., Papatsoris A., Chrisofos M., Dellis A., Liakouras C., Skolarikos A. (2005). Urinary diversion in high-risk elderly patients: Modified cutaneous ureterostomy or ileal conduit?. Urology.

[14] Hollenbeck B.K., Miller D.C., Taub D., Dunn R.L., Underwood W., Montie J.E., Wei J.T. (2004). Aggressive treatment for bladder cancer is associated with improved overall survival among patients 80 years old or older. Urology.

[15] Prout G.R., Wesley M.N., Yancik R., Ries L.A., Havlik R.J., Edwards B.K. (2005). Age and comorbidity impact surgical therapy in older bladder carcinoma patients: A population-based study. Cancer.

[16] Izquierdo L., Peri L., Leon P., Ramírez-Backhaus M., Manning T., Alcaraz A., Rouprêt M., Solsona E., Rubio J., Sengupta S. (2015). The role of cystectomy in elderly patients—A multicentre analysis. BJU Int..

[17] Nogueira L., Reis R., Machado R.D., Tobias-Machado M., Carvalhal G.F., Freitas C., Magnabosco W.J., Menezes C.L., Corradi C., Reis L. (2013). Cutaneous ureterostomy with definitive ureteral stent as urinary diversion option in unfit patients after radical cystectomy. Acta Cir. Bras..

[18] Siddiqui K.M., Izawa J.I. (2016). Ileal conduit: Standard urinary diversion for elderly patients undergoing radical cystectomy. World J. Urol..

[19] Mucciardi G., Macchione L., Gal A., Di Benedetto A., Subba E., Pappalardo R., Mucciardi M., Butticè S., Inferrera A., Magno C. (2015). Quality of life and overall survival in high risk patients after radical cystectomy with a simple urinary derivation. Cir. Esp..

[20] Chang S.S., Alberts G., Cookson M.S., Smith J.A. (2001). Radical cystectomy is safe in elderly patients at high risk. J. Urol..

[21] Liu Z., Meng Y., Li S., Yu W., Jin J. (2020). Perioperative recovery in different urinary reconstruction approaches of radical cystectomy: Are the advantages of laparoscopy consistent?. J. Minim. Access Surg..

[22] Mortezavi A., Crippa A., Edeling S., Pokupic S., Dell’Oglio P., Montorsi F., D’Hondt F., Mottrie A., Decaestecker K., Wijburg C.J. (2020). Morbidity and mortality after robot-assisted radical cystectomy with intracorporeal urinary diversion in octogenarians: Results from the European Association of Urology Robotic Urology Section Scientific Working Group. BJU Int..

[23] Miller D.C., Taub D.A., Dunn R.L., Montie J.E., Wei J.T. (2003). The impact of co-morbid disease on cancer control and survival following radical cystectomy. J. Urol..

[24] Kulkarni J.N. (2011). Perioperative morbidity of radical cystectomy: A review. Indian J. Urol..

[25] Longo N., Imbimbo C., Fusco F., Ficarra V., Mangiapia F., Di Lorenzo G., Creta M., Imperatore V., Mirone V. (2016). Complications and quality of life in elderly patients with several comorbidities undergoing cutaneous ureterostomy with single stoma or ileal conduit after radical cystectomy. BJU Int..

[26] De Nunzio C., Cicione A., Leonardo F., Rondoni M., Franco G., Cantiani A., Tubaro A. (2011). Extraperitoneal radical cystectomy and ureterocutaneostomy in octogenarians. Int. Urol. Nephrol..

[27] Ahmadi H., Lee C.T. (2015). Health-related quality of life with urinary diversion. Curr. Opin. Urol..

[28] Saika T., Arata R., Tsushima T., Nasu Y., Suyama B., Takeda K., Ebara S., Manabe D., Kobayashi T., Tanimoto R. (2007). Health-related quality of life after radical cystectomy for bladder cancer in elderly patients with an ileal conduit, ureterocutaneostomy, or orthotopic urinary reservoir: A comparative questionnaire survey. Acta Med. Okayama.

